# Artificial Induction of Meiotic Gynogenesis in *Koi* Carp Using Blunt Snout Bream Sperm and Identification of Gynogenetic Offspring

**DOI:** 10.3390/ani15101411

**Published:** 2025-05-13

**Authors:** Xiaoyu Chen, Xiulan Shi, Jun Guo, Kai Lin, Mingkun Luo, Zaijie Dong

**Affiliations:** 1 Wuxi Fisheries College, Nanjing Agricultural University, Wuxi 214081, China; chenxiaoyu@stu.njau.edu.cn (X.C.); sxl1253707210@163.com (X.S.); 2 Jiangsu Qihong Ecological Agriculture Development Co., Ltd., Suzhou 215416, China; w17639847628@163.com (J.G.); 13939818948@163.com (K.L.); 3 Key Laboratory of Freshwater Fisheries and Germplasm Resources Utilization, Freshwater Fisheries Research Center of Chinese Academy of Fishery Sciences, Ministry of Agriculture and Rural Affairs, Wuxi 214081, China

**Keywords:** gynogenesis, *koi* carp, microsatellite, chromosome, 5S rDNA

## Abstract

Artificial gynogenesis is an effective means of producing pure lines and is widely used for genetic analysis of fish and for sex control. In this study, inactivated sperm from heterogenous blunt snout bream (*Megalobrama amblycephala*, MA) were used to activate *Kohaku koi* (*Cyprinus carpio* var. *koi*, CK) and produce high-quality female offspring. To determine whether the offspring were gynogenetic fish, the karyotype and DNA content of the CK, MA and the induced offspring (IO) were first compared and it was found that the IO were diploid with 100 chromosomes and their karyotype was 22m + 34sm + 22st + 22t. The DNA content of the IO was not significantly different from that of the CK. Subsequently, the amplified band profiles of CK, MA and IO were analyzed with species-specific microsatellite markers. The results showed that there were no amplified MA microsatellite bands in IO. The size of the amplified bands and the sequence of the 5S rDNA in CK, MA and IO were also analyzed. It was found that the amplified 5S rDNA gene fragments in IO contained two fragments that were both the same size as those of CK and matched more than 90% with those of CK. Finally, the sex of IO was verified using gonadal tissue sections. The result showed that IO was not an all-female population; males were also present (36.7%). In summary, a series of validation methods confirmed that the induced offspring were gynogenetic fish, which is the basis for the subsequent genetic improvement of pure lines of high-quality *koi*.

## Simple Summary

Through the gynogenesis technique, it was obtained a number of female *koi* with high body size and color, which helped to solve the problem of breeding purebred *koi* lines and improving germplasm. To ensure that the offspring obtained were all-female karyotypes, the offsprings were analyzed using a combination of methods, and fully confirmed that the genetic information of the offspring was the same as that of the mother, but there were also a certain number of males in the offspring, which needs to be further pursued and clarified.

## 1. Introduction

The *koi* carp (*Cyprinus carpio* var. *koi*) is one of the most popular ornamental fish in the world [1]. In traditional Chinese culture, the *koi* symbolizes happiness, harmony and success and has important market and cultural value. With the continuous development of the *koi* breeding industry, some problems have gradually come to light, such as the fact that the traditional breeding process requires constant screening to obtain *koi* with high ornamental value. On the other hand, the actual production of *koi* has also shown that females have a broader back and a more robust body than males, and their ornamental value is also higher. Therefore, from the breeders’ point of view, there is an urgent need for technological solutions to improve the efficiency of *koi* breeding, and producing all-female *koi* will help to increase economic returns. Gynogenesis is an important technological method in fish breeding that allows pure lines to be established quickly, effectively fixing the dominant genetic traits and shortening the breeding cycle [2]. This technique has been applied to a number of economically important fish species, including grass carp (*Ctenopharyngodon idella*) [3], tilapia (*Oreochromis niloticus*) [4], Atlantic salmon (*Salmo salar*) [5], rainbow trout (*Oncorhynchus mykiss*) [6], flounder (*Paralichthys olivaceus*) [7], yellow catfish (*Pelteobagurus fulvidraco*) [8], etc.

The offspring of gynogenesis sometimes show characteristics of both the male and the female individual, which could be due to the integration of paternal DNA during gynogenesis, known as the allo-sperm effect [9]. Using heterologous sperm to induce gynogenesis may avoid this phenomenon [10]. As early as in 1992, Komen et al. used inactivated carp spermatozoa to activate gynogenesis in *koi* [11]. In this study, blunt snout bream (*Megalobrama amblycephala*, MA) were used as male tributary spermatozoa, which belongs to a different subfamily and are genetically distant from *koi*, and theoretically the two cannot produce normal hybrid offspring. However, it has also been reported in the literature that the two are capable of distant hybridization to produce a new strain [12]. Therefore, to more accurately determine the characteristics of gynogenetic progeny, morphological methods should be combined with molecular and cell biology methods, such as species-specific molecular markers, karyotyping and DNA content detection [13], to better analyze their biological characteristics. In this study, gynogenesis was induced in *koi* carp using UV-treated MA spermatozoa and a number of offspring were successfully obtained. The induced offspring (IO) were analyzed for gynogenesis by chromosome ploidy, DNA content, microsatellites, 5S ribosomal DNA and tissue sections. The results of the study can help to improve the pedigree of high-quality *koi* and lay the foundation for breeding unisexual *koi*.

## 2. Materials and Methods

### 2.1. Experimental Material

The experiment was conducted in accordance with the Institutional Animal Care and Use Committee (IACUC) of the Freshwater Fisheries Research Center of Chinese Academy of Fishery Sciences (FFRC-CAFS) (No. 398, 2006). The experimental *koi* carp (CK) were obtained from Jiangsu Qihong Ecological Agriculture Development Co., Ltd. (Suzhou, China) and the MA from Nanquan Base of FFRC. All fish used as samples were anaesthetized with 15 mg/L MS-222 (Sigma, St. Louis, MO, USA) before the procedure [14].

### 2.2. Induction of Gynogenetic Offspring

Sperm inactivation treatment: A well-matured male MA (23 cm, 1 kg) was selected as a sperm donor. The semen was collected by gently squeezing the belly of the fish and then mixed with pre-chilled Hank’s solution (Wisent Inc., Montreal, QC, Canada) at a ratio of 1:4. The diluted semen was placed in a petri dish and placed on a shaker illuminated with a UV lamp. The spermatozoa were inactivated by UV irradiation for 10 min. The light intensity of the UV lamp was 52 μW/cm^2^; the distance between the lamp and the semen was 32 cm.

Obtaining eggs and female zygotes: An adult female *Kohaku* (red and white color, 60 cm, 3.7 kg) with optimal body shape was selected as an egg donor and injected with oxytocin. After the hormone took effect, the eggs were squeezed into a basin and MA’s genetically inactivated sperm diluent was immediately added. The sperm and eggs were gently agitated and mixed thoroughly for 2 min, then water was added to initiate egg development. The gynogenetic line was induced by cold shock at 4 ± 0.5 °C for 20 min. The eggs were then placed in water at room temperature (23 ± 1 °C) until hatching.

### 2.3. Karyotyping of Induced Offspring (IO)

Nine IO individuals were randomly selected and injected with phytoheagglutinin (PHA) at a dose of 10 μg/g (fish weight) [15]. After 12 h, 0.5% colchicine was injected into the peritoneal cavity of the fish at a dose of 5 μg/g (fish weight). Three hours later, the kidney cells were collected by filtration through nylon gauze and then subjected to hypotonic treatment with 0.075 mol/L KCl for 30 min at room temperature and fixed with Carnot fixative (methanol: acetic acid = 3:1) for 30 min at 4 °C; this step was repeated three times. Finally, the cell suspension was dropped onto the cold slide, dried at room temperature and then placed in 15% Giemsa staining solution for staining. Then, the slides were dried and photographed. The chromosome number was counted under the microscope [16]. The images were processed using Photoshop 2023 (Adobe Inc., San Jose, CA, USA) and Image J 1.54 (NHI, Bethesda, MD, USA). The parameter measurements of the five metaphase images were analyzed and chromosome karyotype maps were generated according to Levan et al., 1964 [17].

### 2.4. Measurement of DNA Content

The DNA content of CK, MA and IO was measured with a flow cytometer (Cell Counter Analyzer, Partec, Bad Mergentheim, Germany) to determine ploidy. Blood was collected from the caudal vein using a disposable syringe rinsed with anticoagulant sodium citrate (ACD). Subsequently, 1 mL of blood was used to determine the relative DNA content [2]. The DNA content of each sample was measured under identical conditions, using the DNA content of the FFRC No.2 strain common carp as a reference.

### 2.5. Microsatellite DNA Analysis

Thirty IO individuals were randomly selected for species-specific microsatellite DNA detection. Genomic DNA was extracted from the caudal fins of 30 offspring and two parental fish using the Marine Animal Genome DNA Rapid Extraction Kit (TIANGEN; Beijing, China). The quality of DNA was checked by 1% agarose gel electrophoresis and stored in a refrigerator at −20 °C until use.

Based on previous literatures [18,19,20,21,22,23], it was selected 20 pairs of SSR primers (10 from common carp, 10 from blunt snout bream to screen the species-specific SSR markers (Table 1). The above primers were synthesized by Sangon Biotech Co., Ltd. (Shanghai, China).

The PCR amplification system comprised 25 μL volume reaction solution, consisting of 9.5 μL ddH_2_O, 12.5 μL Taq enzyme system (containing 10 × buffer, dNTP, Taq enzyme), 1 μL DNA template and 2 μL primers (10 μmol/L). The amplification procedure consisted of pre-denaturation at 95 °C for 3 min, 30 cycles of denaturation at 95 °C for 15 s, annealing at 58 °C for 15 s, amplification at 72 °C for 15 s and final extension at 72 °C for 5 min. The PCR products were subjected to agarose gel electrophoresis. The screened species-specific SSR markers were used to identify the IO.

### 2.6. Amplification of 5S rDNA

Based on the literature [24,25], it was designed two pairs of 5S rDNA primers (primer 1, forward sequence: 5′-GCTATGCCCGATCTCGTCTGA-3′, reverse sequence: 5′-CAGGTTGGTATGGCCGTAAGC-3′; primer 2, forward sequence: 5′-TATGCCCGATCTCGTCTGATC-3′, reverse sequence: 5′-CAGGTTGGGTATGGCCGTAAGC-3′) for PCR amplification. The reaction system comprised 25 μL volume with the following conditions: pre-denaturation at 95 °C for 5 min, 30 cycles of denaturation at 95 °C for 10 s, annealing at 56 °C for 10 s and extension at 72 °C for 5 min. The PCR products were separated by 1.5% agarose gel electrophoresis, and then ligated into the pMD18-T vector after purification. A single colony was selected for Sanger sequencing. The sequences were analyzed using MEGA 11.0 software (NVIDIA Corporation, Santa Clara, CA, USA).

### 2.7. Sex Identification of IO

Thirty 11-month-old IO individuals with an average body weight of 38.57 g were randomly selected for dissection of gonadal tissues. IO gonadal tissues were fixed with 4% paraformaldehyde, dehydrated in a series of ethanol dilutions, passed through xylene, and embedded in paraffin. The tissue was then sectioned at 5 μm thickness and mounted on slides. The slides were stained with hematoxylin–eosin (HE) and observed under a light microscope (OLYMPUSBX51, Olympus Corporation, Tokyo, Japan). The sex of the IO was identified based on the observation.

## 3. Results

### 3.1. Number of Surviving Offsprings

There were 734 surviving IO individuals with three phenotypes, of which 294 were red-white colored (RW), 308 were pure red colored (WR) and 132 were pure white colored (WW). This result showed that only 40% of the offspring had the same color as the female parent.

### 3.2. Chromosome Numbers and Karyotypes of IO

**Figure 1:**
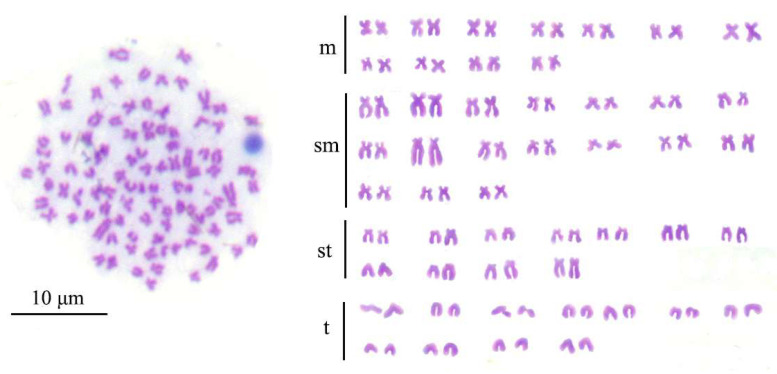
Chromosome number and karyotype of IO, m: metacentric; sm: submetacentric; st: subtelocentric; t: telocentric.

Based on the observation of chromosome slides of IO (Figure 1), most IO were found to have 100 chromosomes, indicating that IO was 2n = 100, the same as CK. The karyotype of IO was 22m + 34sm + 22st + 22t. The karyotype of *koi* was 22m + 34sm + 22st + 22t [26], while the karyotype of blunt snout bream was 18m + 26sm + 4st [27]. The above results showed that the chromosome numbers and karyotypes of IO were identical to those of CK.

### 3.3. DNA Content of IO

The DNA content of MA, CK and IO (including WR, WW and RW) was determined by flow cytometry using the FFRC No.2 strain common carp (2n = 100) as a reference. The average DNA content of MA, CK and IO (WR, WW, RW) was 28.12, 37.59, 36.39, 36.52 and 36.57, respectively (Table 2). Comparing the ratio of DNA content between IO and CK (0.97) and between IO and MA (1.3), it could be concluded that the ploidy of IO was the same as that of CK.

### 3.4. Microsatellite DNA Analysis of IO

**Figure 2:**
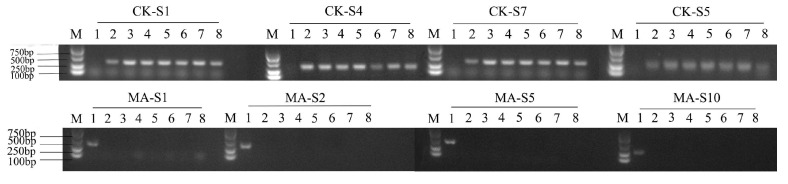
The four screened-out pairs of common carp-specific SSR primers (CK-S1, CK-S4, CK-S7, CK-S5) and four pairs of blunt snout bream-specific SSR primers (MA-S1, MA-S2, MA-S5, MA-S10). Lane 1: MA, Lane 2: CK, Lanes 3-8: IO.

**Figure 3:**
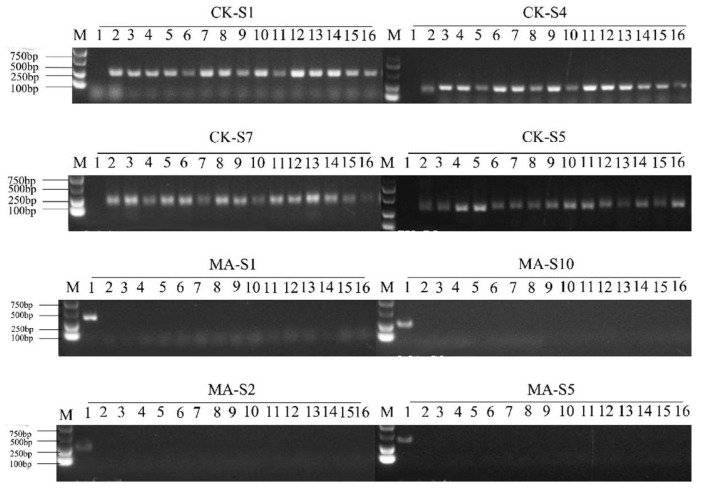
The amplification band profiles of IO with common carp-specific SSR primers (CK-S1, CK-S4, CK-S7, CK-S5) and blunt snout bream-specific SSR primers (MA-S1, MA-S2, MA-S5, MA-S10). Lane 1: MA, Lane 2: CK, Lanes 3–16: IO.

First, four pairs of common carp-specific SSR primers and four pairs of blunt snout bream-specific SSR primers were screened out from the above 20 primers (Figure 2). Then, 24 IO individuals were randomly selected and amplified with these eight primer pairs. When amplified with blunt snout bream-specific primers, the amplification bands appeared only in MA. When amplified with carp-specific primers, the amplification bands were found in both CK and IO, but no bands appeared in MA (Figure 3).

### 3.5. Molecular Organization of 5S rDNA

**Figure 4:**
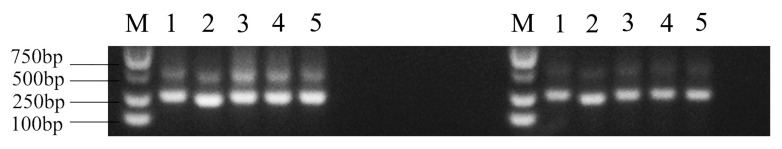
PCR amplification map of 5S rDNA in CK, MA and IO, Lane 1: CK, Lane 2: MA, Lanes 3–5: IO.

**Figure 5:**
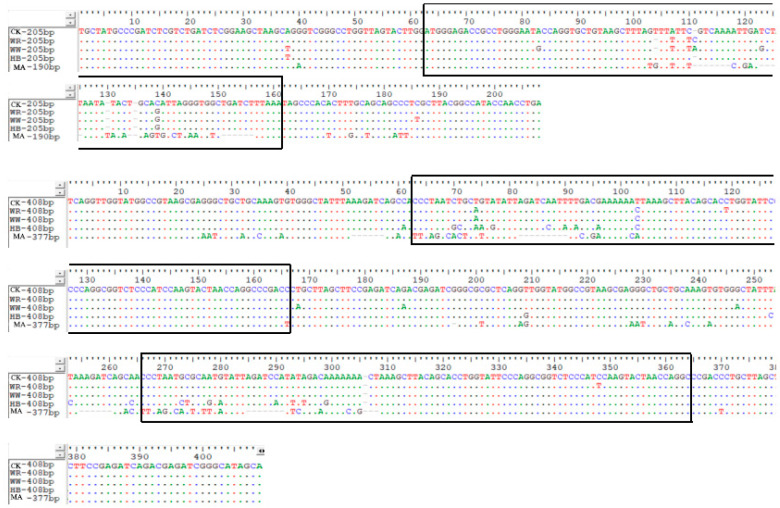
Comparison of complete 5Sr DNA structure amplified from primer 1 among CK, MA and IO (WR, WW, RW). Dots indicate identical nucleotides (the black box shows the CDS area), red is T, green is A, blue is C, and black is G.

**Figure 6:**
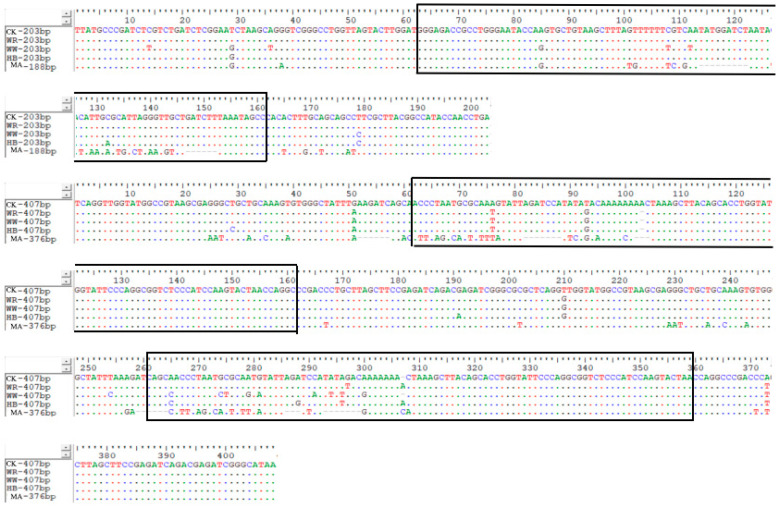
Comparison of complete 5Sr DNA structure amplified from primer 2 among CK, MA and IO (WR, WW, RW) fish. Dots indicate identical nucleotides (the black box shows the CDS area), red is T, green is A, blue is C, and black is G.

The 5S rDNA of CK, MA and IO were amplified and sequenced. Agarose gel electrophoresis results showed that two bands were obtained in all fish (Figure 4). The two band sizes in IO were the same as those in CK. The sequencing results of the amplification products of primer 1 showed that the lengths of the two fragments in CK were 205 bp and 408 bp, respectively; the lengths of the two fragments in MA were 190 bp and 377 bp, respectively (Figure 5). The lengths of the two fragments in IO were the same as those in CK. The sequencing results of the products amplified with primer 2 gave the same result as primer 1 (Figure 6). The two monomeric 5S rDNA classes in CK were class I: 205 bp and class II: 408 bp. The two monomeric 5S rDNA classes in MA were class III: 190 bp and class IV: 377 bp. In IO, there were two classes of monomeric 5S rDNA (class V: 205 bp; class VI: 408 bp) corresponding to those in CK. The 5Sr DNA of IO showed a high degree of similarity to that of CK both in the conserved coding sequence (CDS) and in the variable non-transcribed spacer region (NTS), with a similarity of more than 90%, even up to 100%.

### 3.6. Sex Ratio in IO

**Figure 7:**
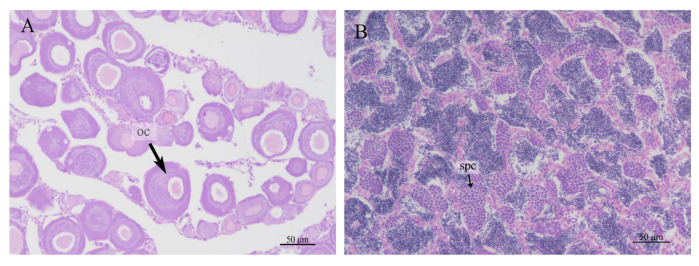
Gonad sections of IO, (**A**): ovary (oc: oocyte), (**B**): testis (spc: spermatocytes).

A total of 30 gonadal tissue samples were collected from the offspring undergoing gynogenesis, and HE staining sections revealed that there were 19 females and 11 males (Figure 7). A certain number of oocytes in developmental stages II and III in the ovary could be clearly recognize on the slides (Figure 7A). A large number of evenly distributed spermatocytes were found in the testis (Figure 7B). This meant that the induced *koi* carp offspring were not all female; the sex ratio of females to males was 1: 0.57. The proportion of males in the IO was 36.7%.

## 4. Discussion

Artificially induced gynogenesis facilitates the rapid production of pure lines, which can theoretically be achieved within two generations, ensuring complete homozygosity of all gene loci. In addition, parents in pure lines can stabilize hybrid dominance [15]. In some species, females are characterized by a faster growth rate and higher commercial value, so a large number of all-female offspring produced by a combination of artificial gynogenesis and artificial sex reversal may be of greater benefit to farmers [28]. Two important steps in the artificial induction of gynogenesis are the inactivation of the genetic material of the sperm and the diploidization of the oocyte. If the sperm’s genetic material is not completely inactivated during artificial induction of gynogenesis in fish, it will lead to fertilization of the eggs [29]. If the time of temperature shock cannot diploidize the oocyte, the eggs from gynogenesis will not survive [30]. Therefore, it is essential to verify the authenticity of the offspring produced by artificially induced gynogenesis. In this study, gynogenesis was induced in *koi* carp using UV-inactivated blunt snout bream spermatozoa, and the induced offspring were subjected to various validation methods, including chromosomal karyotyping, DNA content determination, microsatellite marker detection, and 5S rDNA sequencing, to confirm that they were gynogenetic.

Determining the ploidy of the induced offspring can be an effective and direct method to determine the success of gynogenesis. Commonly used methods to determine ploidy include relative karyotype analysis and DNA content determination [31]. Chromosomes serve as carriers of genetic information and are the most direct and accurate evidence for determining the ploidy of gynogenetic offspring. Chromosome karyotype analysis is now widely used to identify gynogenesis. The karyotype of IO in this study was 22m + 34sm + 22st + 22t, which means that the chromosome number of IO is 100. This result is consistent with the karyotype of *koi* carp described by Wang et al. [26]. The findings confirm that the gynogenetic offspring’s chromosome karyotype is identical to that of the mother, indicating that the sperm merely serves as a developmental stimulus and does not contribute to the genetic material of the offspring The determination of the relative DNA content by flow cytometry allows for a fast and direct assessment of the ploidy of fish chromosomes. Currently, flow cytometry is widely used for ploidy identification in both animals and plants [13,32]. In this study, the detection of DNA content revealed that the relative ratio of DNA content of IO to CK was about 0.97, while the ratio of IO to MA was about 1.3. This indicates that the DNA content of IO is essentially the same as that of the mother fish.

There are various methods for identifying gynogenesis. Molecular marker technology is the most reliable and convenient method for detecting gynogenesis in fish [33,34]. Currently, the most commonly used molecular markers are AFLP and microsatellite markers. In this study, eight pairs of species-specific microsatellite primers were screened and used to amplify microsatellite markers in CK, MA and IO. The result showed that the amplified bands in IO matched those in CK, indicating that the genetic information of the induced offspring was essentially the same as that of the female parents. This is consistent with the results of Xie et al. [35], who selected four pairs of microsatellite primers for amplification in the gynogenetic offspring of *Nibea albiflora*, and the results showed that all the offspring did not contain the paternal genes. However, to fully validate the gynogenetic offspring, as many SSR markers as possible are needed to cover most of the genomic information.

5S rDNA consists of simple tandem repeat units, including a 120 bp length, highly conserved coding sequence (CDS) and a variable non-transcribed spacer (NTS) region. The NTS region is known for its high variability, which includes insertions, deletions, duplications and base substitutions. Due to the high variability of the NTS region in 5S rDNA, it is therefore a valuable tool for species evolution studies and is used as a species-specific or population-specific molecular marker [24]. Mao [15] analyzed the 5S rDNA genes in the gynogenetic grass carp population and found that their NTS region was identical to that of grass carp and had minimal similarity to that of *koi* carp. The CDS and NTS regions of the IO in this study were highly conserved with those of the CK, indicating that the 5S rDNA sequences of the induced offspring were inherited from the *koi* carp.

The mechanism of sex determination in fish is complex and is influenced not only by genetic factors but also by environmental conditions. Temperature is a very important factor in the environmental conditions of sex reversal in fish [36]. Therefore, understanding the mechanisms of sex determination in fish has fascinated scientists. Gynogenesis is a method of producing monosexual groups in species with XX/XY sex determination systems, and theoretically, the gynogenetic offspring should be exclusively female, as both sex chromosomes are inherited exclusively from the mother. However, it has been observed that some fish species produce male individuals or even a significant proportion of males after gynogenesis. For instance, genetic analysis of sex reversal in gynogenetic rainbow trout revealed a male-to-female ratio of approximately 1:1 [37], while gynogenetic tilapia have a male proportion of up to 35.3% [38]. Gynogenetic zebrafish (*Danio rerio*) also predominantly produce males [39]. In this study, the IO was not purely female; males also occurred, which is consistent with these findings. Komen et al. [11] found that a recessive mutation of an autosomal sex-determining gene (*mas*-*l*) could lead to three phenotypes (female, male and intersex) in the sexual differentiation of common carp. Maybe a similar mechanism caused the appearance of males in the current study. However, the above study was conducted using common carp as male sperm donors, whereas blunt snout bream was used as male tributes in our study, and we hypothesized that this was due to either a lack of inactivation of genes encoding testis development in sperm or mutations in genes associated with sex differentiation on the autosomal chromosomes of *koi* carp under unfavorable conditions.

## 5. Conclusions

In this work, we induced gynogenesis of *koi* carp with inactivated sperm of *M. amblycephala*, and the induced offspring were comprehensively identified by chromosomal karyotype, DNA content, microsatellite markers, and 5S rDNA sequence. The offspring were determined to be gynogenetic *koi* carp. This indicates a potential breeding method to produce pure lines of *koi* carp, which may increase the acquisition rate of high-quality individuals. However, there were some males among the gynogenetic offspring. Therefore, the breeding of all-female *koi* carp needs further research.

## Figures and Tables

**Table 1:** Sequence information of 20 primer pairs for CK (*Cyprinus carpio* var. *koi*) and MA (*Megalobrama amblycephala*) analysis.

Primer Name	Sequence Features	Annealing Temperature/°C	GenBank Number
CK-S1	F: TCCAAGTCAGTTTAATCACCG	60	XM_042754805.1
	R: GGGAAGCGTTGAGAACAAGC		
CK-S2	F: GAATCCTCCATCATGCAAAC	57	—
	R: GCACAAACTCCACATTGTGCC		
CK-S3	F: GCTCCAGATTGCACATTATAG	64	XM_042765836.1
	R: CTACACACACGCACAGCCTTTC		
CK-S4	F: AGACCACCGCAGTAACAA	53	XM_042716969.1
	R: GACTCACTCAGCACCAGA		
CK-S5	F: GTACAGCGTGACAGCATT	53	XM_042749979.1
	R: AAGTTCATCGGTGTCCTC		
CK-S6	F: ATCATTTGTATTCGTGCTTG	53	XM_042745909.1
	R: GATCCACTGGGTCCTTTT		
CK-S7	F: CACGACGTTGTAAAACGACTTGT	54	XM_042767288.1
	R: ATTGGTGCAGAGCATCAGTG		
CK-S8	F: CACGACGTTGTAAAACGACGGGG	57	—
	R: CGGCGACTTGATCCTCTTTA		
CK-S9	F: CACGACGTTGTAAAACGACACTA	56	—
	R: CTTGTACCTGCACAGTCTCATC		
CK-S10	F: CACGACGTTGTAAAACGACCCAA	61	XM_042723695.1
	R: AACAAGCATGTAGGCACTA		
MA-S1	F: TGGAGTTAGTGTCCGCTTGT	56	XM_051888809.1
	R: AGGATACGGGTGAGTTCG		
MA-S2	F: TTCGGTTCTGCCTTCACTCT	65	XM_048170375.1
	R: AAGACGCATGCTCAACAAC		
MA-S3	F: GTCCAGACTGTCATCAGGAG	60	XM_048208277.1
	R: GAGGTGTACACTGAGTCACGC		
MA-S4	F: TCAGCTGAGGGATGGATGGA	55	XM_048163853.1
	R: AAGGGAGGCTCAGTGTTTCG		
MA-S5	F: GAGCTCCTCAGAAGGGCTTC	57	XM_048163362.1
	R: CTTTGGGTTCCGTCGACTGA		
MA-S6	F: GATAGTGAGCACGAGCAGGA	57	XM_048211096.1
	R: CCCAGCATGCTTTGTGTAGG		
MA-S7	F: GACTGGAGTCGTCAGGCTTC	60.5	—
	R: TGCCCCACATTGTTAGACTG		
MA-S8	F: GGGGAAATAAAGGGAGAAAGTG	60.5	XM_048195326.1
	R: TTTCTCCTGATCCGTTGACC		
MA-S9	F: AAACAGGCTCGCCAATTTC	55.9	XM_048202478.1
	R: TCACCCACACACTCTTATTCTCT		
MA-S10	F: AGGCGAAAGAAACACTGTGT	56	XM_048178383.1
	R: GGTGTTCGTGCGATGTTGTA		

Note: SSR primers of common carp: CK-S1 to CK-S10; SSR primers of blunt snout bream: MA-S1 to MA-S10. “—” stands for primer sequence information from the references.

**Table 2:** Average DNA content statistics.

Fish		Average DNA Content	Actual Ratio		Theoretical Ratio
			Ratio to CK	Ratio to MA	
CK		37.59 ± 1.188	\	\	\
MA		28.12 ± 0.796	\	\	\
IO	WR	36.39 ± 0.576	0.97	1.3	1
	WW	36.52 ± 0.935			
	RW	36.57 ± 0.577			

Note: CK: *Cyprinus carpio* var. *koi*, MA: *Megalobrama amblycephala*, IO: induced offspring, WR: whole red, WW: whole white, RW: red-white.

## Data Availability

Data is contained within the article.
